# Supplementary material for: The Increase in the Drug Resistance of Acute Myeloid Leukemia THP-1 Cells in High-Density Cell Culture Is Associated with Inflammatory-like Activation and Anti-Apoptotic Bcl-2 Proteins
Source: Int J Mol Sci. 2022 Jul 17;23(14):7881. doi: 10.3390/ijms23147881 (PMC9324792; doi:10.3390/ijms23147881)
Supplement: Supplementary file 1 [file ijms-23-07881-s001.zip › ijms-1772609-supplementary.pdf]

Supplementary Materials

(a)

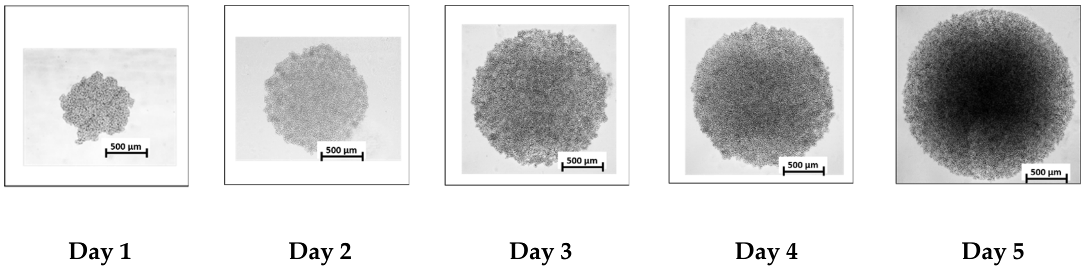

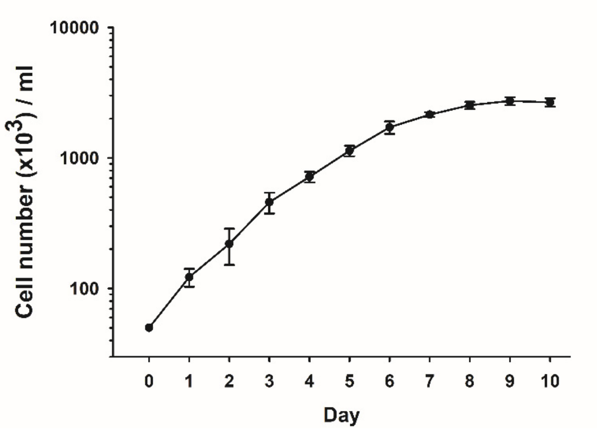

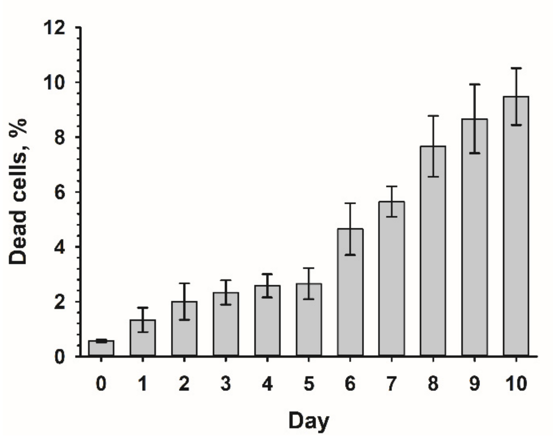

(b) (c)

Figure S1. Micrographs of THP-1 cell cultures during their growth in the first 5 days after seeding of 5000 cells in a U-shaped well. Bar: 500 µm. Growth curve of THP-1 cell culture (**b**) and the percent of dead cells in culture during the growth (**c**).

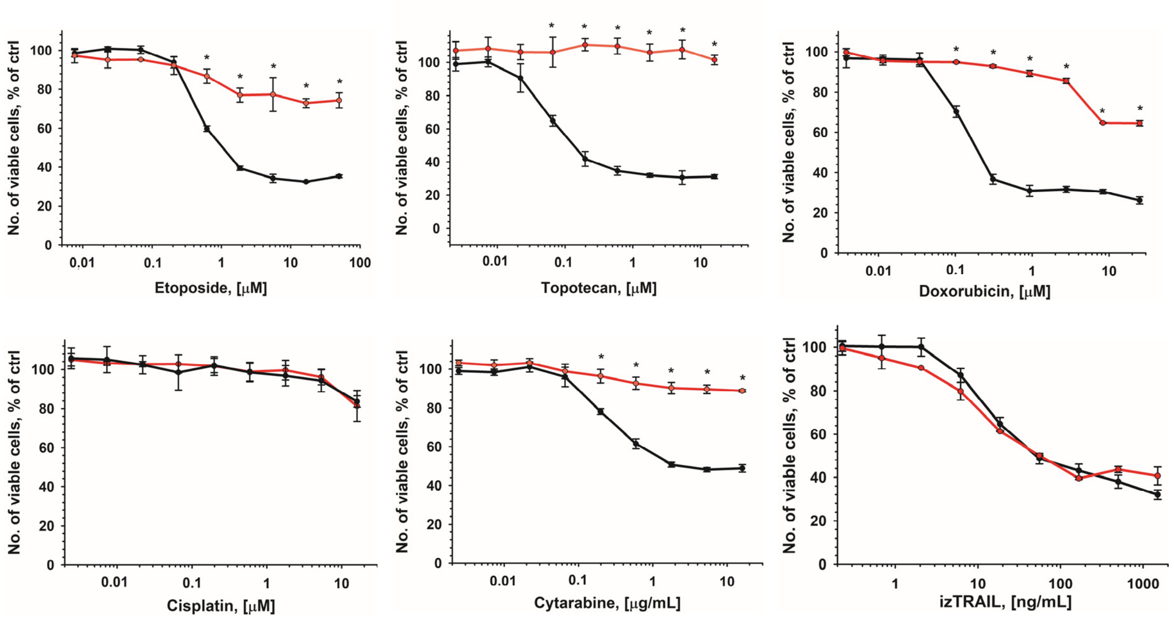

Figure S2. Cytotoxic effect of izTRAIL and different drugs added to HL-60 cell cultures at 1 day (black line) and 5 days (red line) after cell seeding. Data are presented as mean ± standard deviation (n≥3). * $p < 0.05$, compared to HL-60 cell cultures in 1 day.

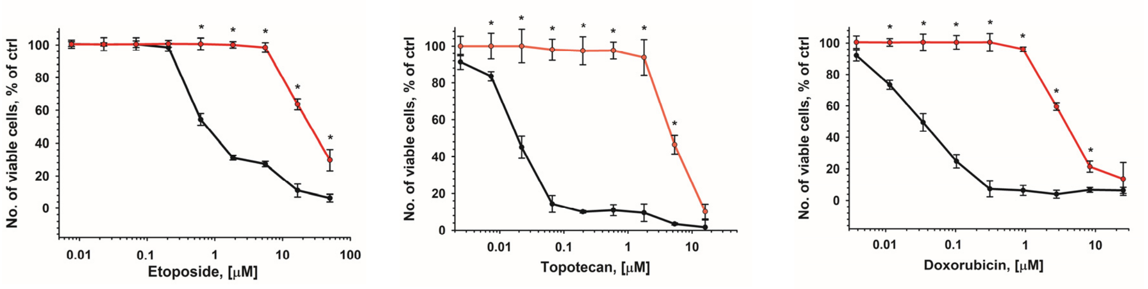

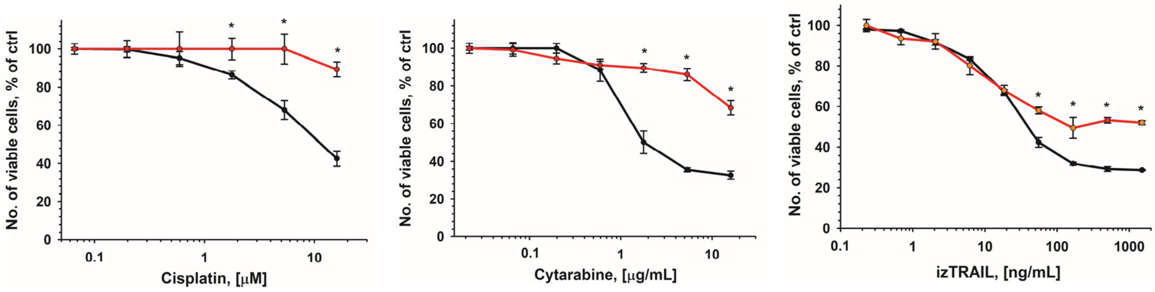

Figure S3. Cytotoxic effect of izTRAIL and different drugs added to MV4-11 cell cultures at 1 day (black line) and 5 days (red line) after cell seeding. Data are presented as mean ± standard deviation (n≥3). * $p < 0.05$, compared to MV4-11 cell cultures in 1 day.

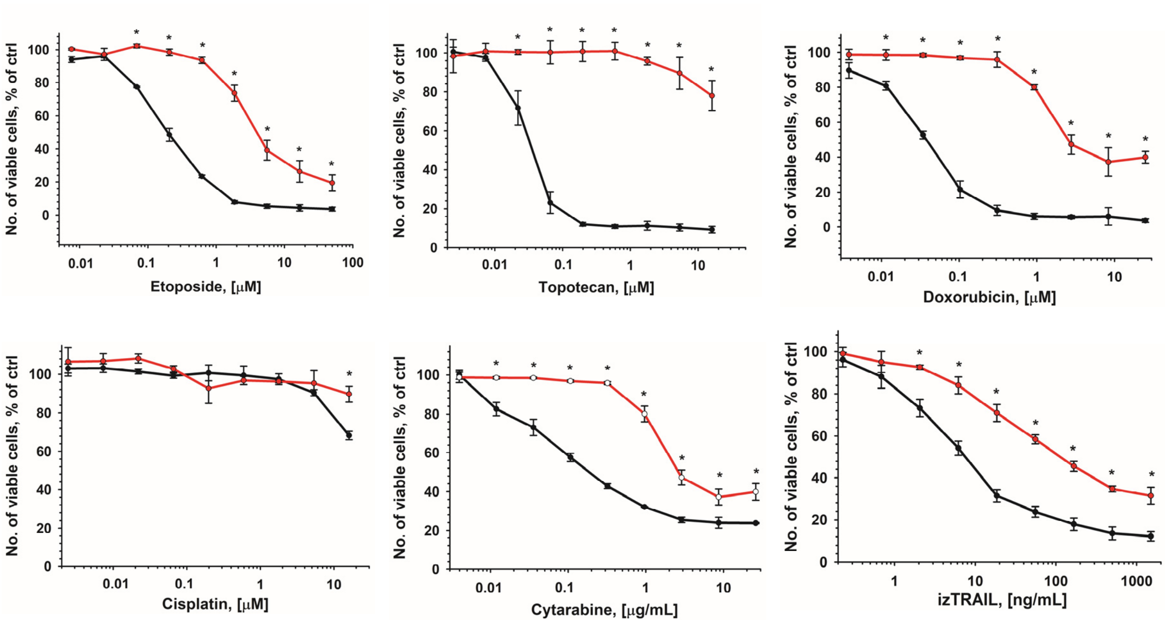

Figure S4. Cytotoxic effect of izTRAIL and different drugs added to U937 cell cultures at 1 day (black line), and 5 days (red line) after cell seeding. Data are presented as mean ± standard deviation (n≥3). * $p < 0.05$ compared to U937 cell cultures in 1 day.

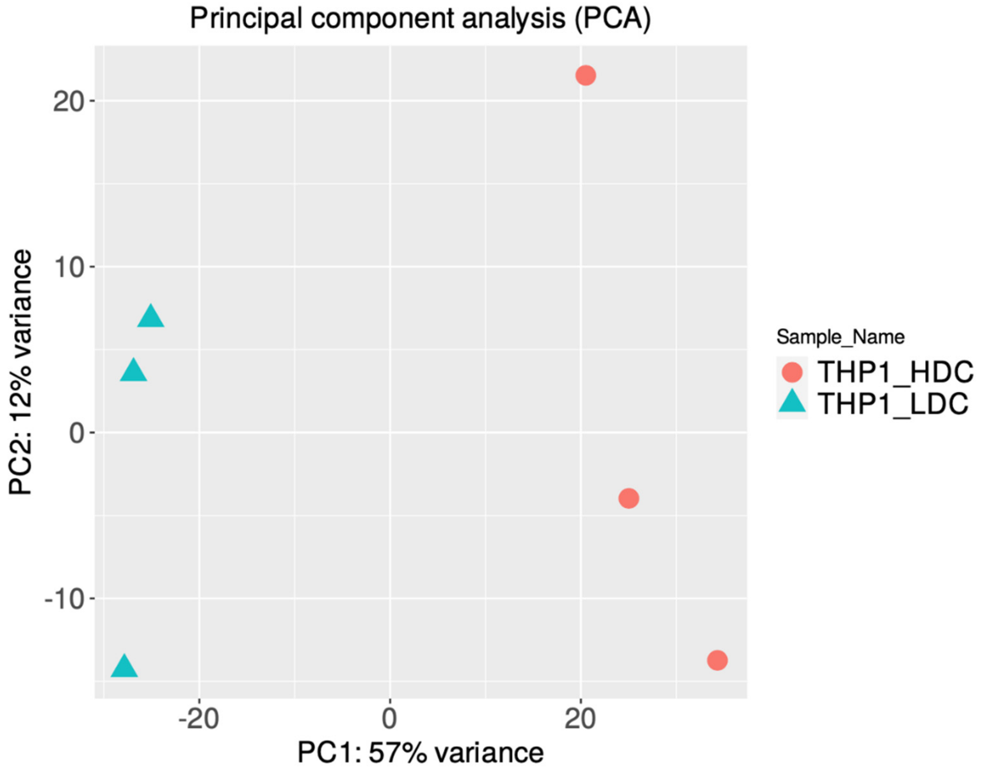

Figure S5. Principal component analysis (PCA) for two groups—THP-1LDC and THP-1HDC in the coordinates of principal component 1 (PC1) and principal component 2 (PC2). n=3 for each group.

Table S1. Differentially expressed genes (DEG) of THP-1HDC cells, compared to THP-1LDC cells ($Log_2FC \geq 1$, $Log_2FC \leq -1$, $p<0.05$).

NAME	log2FC	NAME	log2FC	NAME	log2FC
**MT1G**	-2.673292918	**CREBRF**	1.033918313	**RUFY4**	1.194376081
**MT2A**	-1.859035436	**SCD**	1.04152174	**PPFIA4**	1.212961461
**MT1E**	-1.583971162	**GNG7**	1.047113929	**ARL4C**	1.237101689
**GAL**	-1.500150535	**SLC9A7**	1.047977605	**COL9A2**	1.240389703
**RGS16**	-1.481704527	**NOP53**	1.048085976	**MYLIP**	1.245911168
**SLC7A5**	-1.464930232	**HSH2D**	1.049802981	**N4BP2L1**	1.251799491
**CD1D**	-1.437369466	**SLC37A2**	1.050910694	**TNF**	1.261110771
**MT1X**	-1.316147032	**BNIP3L**	1.058338052	**FADS1**	1.26228806
**SLC30A1**	-1.293095248	**DDIT4**	1.058916216	**CYBB**	1.267068338
**ARRDC4**	-1.27888602	**GIPC3**	1.061129645	**SDK2**	1.271901614
**SAMD11**	-1.263399519	**C1orf228**	1.064941298	**LDLR**	1.301157687
**CD320**	-1.258605578	**ALDOC**	1.066344487	**HIP1**	1.313726161
**MAP1B**	-1.253641845	**CLIP2**	1.069613	**LSP1**	1.327364517
**TLN2**	-1.160119308	**XYLT1**	1.071258984	**INSIG1**	1.355498208
**KCNK5**	-1.117704613	**CACNA2D4**	1.074251364	**CD209**	1.365631512
**NLN**	-1.110228923	**CX3CR1**	1.076556852	**BMF**	1.372732581
**ODC1**	-1.064003454	**PRLR**	1.077248824	**SULF2**	1.426014908
**TRMT61A**	-1.052858193	**ABTB1**	1.077902363	**RAB37**	1.430299818
**ID3**	-1.051754157	**SLAMF7**	1.080117863	**SLC44A2**	1.448042371
**PRADC1**	-1.050452163	**LY86**	1.082216476	**RASGRP4**	1.449425188
**FKBP4**	-1.045754801	**TNFAIP3**	1.084784159	**SH2D3C**	1.476696833
**CCDC86**	-1.042483453	**MPEG1**	1.093231745	**VSIR**	1.482963513
**ID1**	-1.029890874	**PTGS1**	1.10000146	**RASSF4**	1.491612012
**MT1F**	-1.029680898	**ADGRA2**	1.100855683	**YPEL3**	1.527142603
**PYCR3**	-1.024595041	**HMX3**	1.102594547	**EPAS1**	1.572181021
**IPO4**	-1.017335363	**YPEL5**	1.102914805	**DNAJC5B**	1.60240274
**POLR3G**	-1.007739638	**ETS2**	1.104228052	**IL7R**	1.64604079
**RAB3IL1**	-1.006222418	**TIMP3**	1.115829584	**C6orf223**	1.650117623
**NPM3**	-1.002785842	**F13A1**	1.117008274	**ABCA1**	1.687903624
**CD37**	1.003189914	**KLHL24**	1.117846262	**MYO1G**	1.747730076
**CCNG2**	1.003902137	**NCOA3**	1.121935607	**FAM129C**	1.787757336
**PLXNC1**	1.00616129	**METTL7A**	1.122633504	**FCER1G**	1.818516103
**EVI2A**	1.006305557	**DNAH1**	1.122635016	**SREBF1**	1.835649169
**VENTX**	1.007574629	**FOXO4**	1.127891002	**VAT1L**	1.870515867
**TTLL3**	1.008139218	**POU6F1**	1.13835483	**ITGAX**	1.899674964
**LMTK3**	1.010503476	**SECTM1**	1.140299485	**BTG2**	1.964295274
**DOK3**	1.011349184	**FOS**	1.144208888	**FADS2**	2.198012062
**CSMD3**	1.012609268	**P2RX1**	1.156721402		
**FAM20C**	1.016540759	**KLF12**	1.167426254		
**C6orf48**	1.01672642	**HCK**	1.17484792		
**PBXIP1**	1.020240727	**RTN2**	1.18246589		
**LRMP**	1.025362803	**SPEF2**	1.189692632		

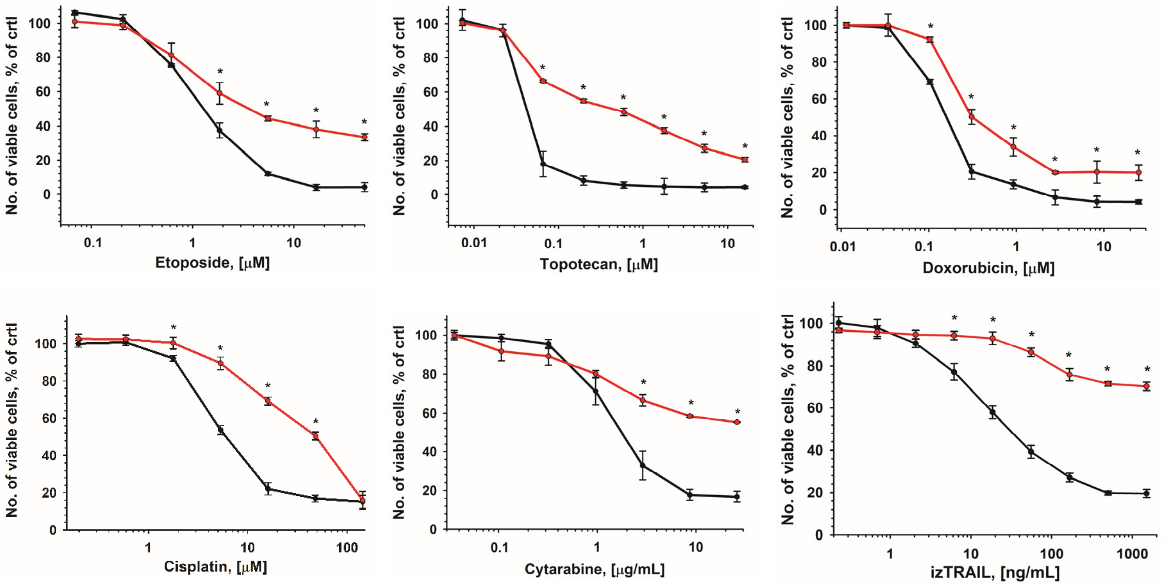

Figure S6. Cytotoxic effect of izTRAIL and different drugs added to THP-1LPS (red line) and THP-1LDC (black line) cells. Data are presented as mean ± standard deviation (n≥3). * - p <0.05, compared to THP-1LDC cells.

Table S2. The percentage of THP-1LDC, THP-1HDC, and THP-1LPS cells bearing CD markers on their surface. Data are shown as mean ± standard deviation (n=3).

CD markers	THP-1LDC	THP-1HDC	THP-1 -LPS
Co-receptor for LPS ***(CD14)***	-	-	**12±1%**
SCARB3 ***(CD36)***	-	**-**	**10±1%**
ICAM-1 ***(CD54)***	-	-	**53±1%**
TLR4 ***(CD284)***	-	-	**14±4%**

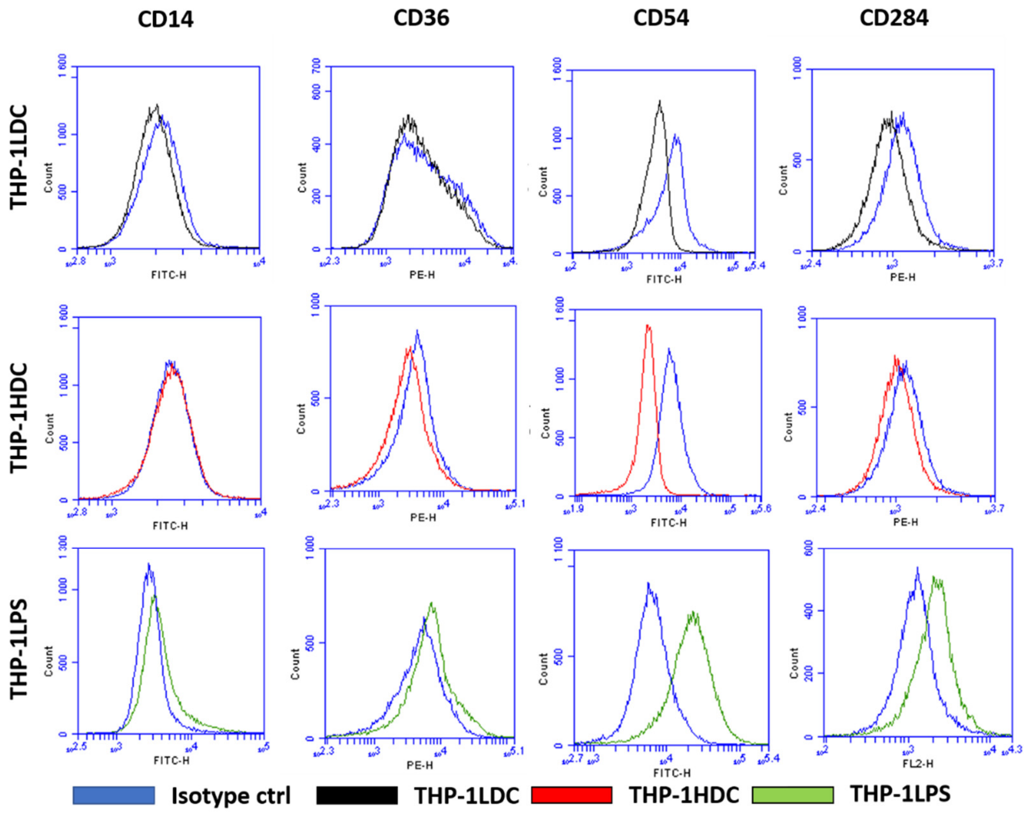

Figure S7. Histograms of expression of the pro-inflammatory markers CD14, CD36, CD54, and CD284 on THP-1LDC, THP-1HDC, and THP-1LPS cells.

Table S3. The concentration of cytokines in growth media of THP-1LDC, THP-1HDC, and THP-1LPS cell cultures, normalized to 1 × 10^5 cells/mL. Data are given as mean ± standard deviation (n=3).

Cytokine	Concentration, pg/mL		
	THP-1LDC	THP-1HDC	THP-1LPS
**IL-1β**	0.22±0.07	0.72±0.06	6.9±0.4
**IL-1ra**	37±6	139.5±9.0	436±18
**IL-2**	0.6±0.1	0.37±0.05	12±1
**IL-4**	-	**0.0231±0.0018**	**0.52±0.04**
**IL-5**	1.8±0.4	1.0±0.1	15.8±0.4
**IL-6**	0.3±0.001	0.9±0.04	101±4
**IL-7**	1.6±0.3	0.3±0.1	3.1±0.1
**IL-8**	1.6±0.2	0.9±0.1	8±1
**IL-9**	2.1±0.3	34.2±2.0	3206±220
**IL-10**	2.0±0.4	0.21±0.03	2.6±0.2

**IL-12p70**	0.3±0.1	0.09±0.02	1.8±0.4
**IL-13**	0.4±0.1	0.06±0.01	0.8±0.1
**IL-15**	17±5	5.3±0.5	56±2
**IL-17A**	0.9±0.2	0.53±0.02	11±1
**Eotaxin**	-	**0.031±0.005**	**0.86±0.03**
**Basic FGF**	-	**1.5±0.1**	**28±1**
**G-CSF**	-	**4.9±0.6**	**117±8**
**GM-CSF**	-	**0.15±0.01**	**2.1±0.1**
**IFN-gamma**	0.6±0.1	0.42±0.02	7.5±0.2
**IP-10**	-	**650.3±67.1**	**1836±137**
**MCP-1**	0.5±0.1	30.5±1.1	5063±2311
**MIP-1α**	-	2.8±0.3	31±4
**MIP-1β**	2.1±0.3	418±18	733±65
**PDGF-BB**	5±1	18±2	28±2
**Rantes**	41±5	514±41	155±12
**TNF-α**	3±1	57±2	364±12
**VEGF**	27±5	88±11	103±9

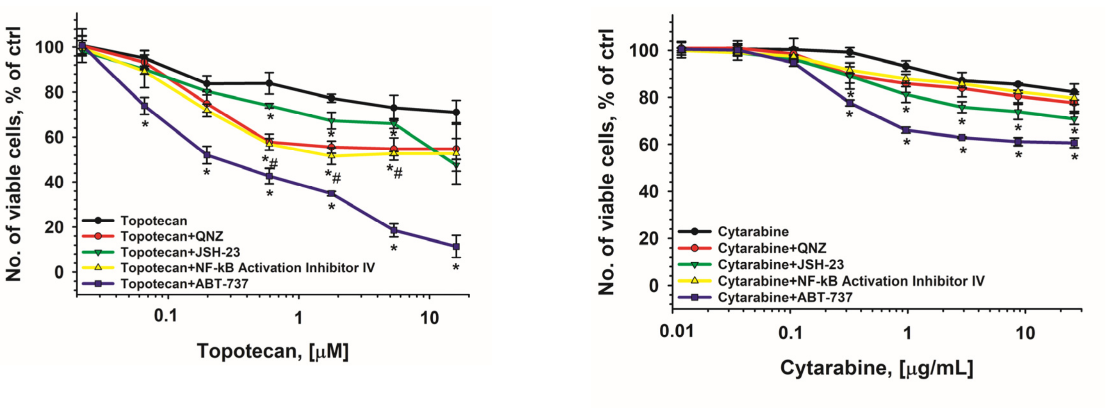

(a) **(b)**

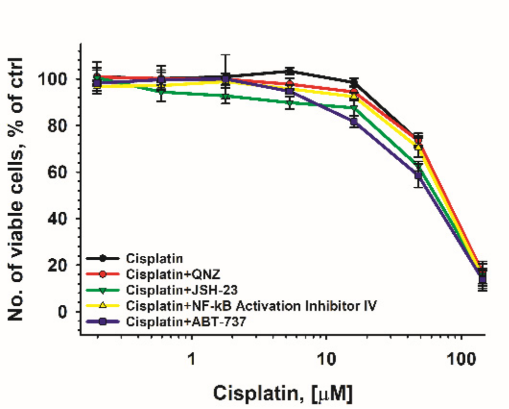

(c)

Figure S8. Effect of the inhibitors of NF-kB (JSH-23 (100 nM), QNZ (40 μM), and NF-kB Activation Inhibitor IV (50 nM)) and of the anti-apoptotic proteins of the Bcl-2 family (ABT-737, 3 μM) on increased resistance of THP-1HDC cells to topotecan (**a**), cytarabine (**b**), and cisplatin (**c**). Data are presented as the mean ± standard deviation. (n≥3). *, # $p<0.05$, compared to control (cells treated with DNA-damaging agent only).
